# Use of infrared thermography in patients undergoing primary amputation due to peripheral arterial obstructive disease

**DOI:** 10.1590/0100-6991e-20243733-en

**Published:** 2024-11-07

**Authors:** LEONARDO MARTINS MOTA DE MORAIS, CLÁUDIO OLAVO CORDOVA, ESDRAS MARQUES LINS, ANA PAULA DE LIMA FERREIRA, VITTORIA MELO LETTIERI, FERNANDA APPOLONIO ROCHA, EMMANUELLE TENÓRIO A. GODOI BERENGUER DE BARROS SILVA

**Affiliations:** 1 - Hospital das Forças Armadas, Cirurgia Vascular - Brasília - DF - Brasil; 2 - Universidade Federal de Pernambuco, Programa de Pós Graduação em Cirurgia - Recife - PE - Brasil; 3 - Universidade de Brasília, Programa de Pós-Graduação em Ciências Médicas - Brasília - DF - Brasil; 4 - Universidade Federal de Pernambuco, Programa de Pós-Graduação em Fisioterapia - Recife - PE - Brasil

**Keywords:** Amputation, Peripheral Arterial Disease, Thermography, Amputação Cirúrgica, Doença Arterial Obstrutiva Periférica, Termografia

## Abstract

**Introduction:**

Peripheral Arterial Disease (PAD) is highly prevalent and the final stage of the disease is the Critical Ischemia (CI) of the Lower Limbs (LL), culminating, in most cases, with amputation of the limbs as part of the proposed treatment. Infrared Thermography (IT) is an inexpensive method, painless, without emission of radiation and easy to manage, which aims to determine the temperature of the skin of the limb to be amputated, and could help the surgeon to evaluate the level of the lower limb amputation.

**Objective:**

To Evaluate Whether IT is a useful method to determine the level of lower limb amputation in patients with PAD and CI. Method: Prospective cohort study performed from April 2023 to November 2023, at the Unit of vascular surgery - Hospital de Base do Distrito Federal (Brasília-DF). It evaluated patients with PAD and CI that were in the preoperative period for lower limb amputation. All Patients Underwent IT evaluation standards protocols.

**Results:**

The results showed a wider longitudinal thermal gradient in PAD smoking (S) patients compared to non-smokers. It was also observed that (S) patients with wide thermal gradients were more likely to undergo an above the knee amputation.

**Conclusion:**

Infrared thermography was a useful method in discriminating thermal differences in patients with PAD and CI could be employed in preoperative evaluation to choose the level of lower limb amputation. Smoking patients with greater longitudinal thermal gradients are more likely to undergo an above the knee amputations.

## RESUMO

**Introdução:**

A isquemia crítica (IC) dos membros inferiores (MMII) é o estágio final da Doença Arterial Obstrutiva Periférica (DAOP) e termina muitas vezes na amputação. A decisão do melhor nível de amputação frequentemente é baseada no julgamento clínico do médico cirurgião vascular. A Termografia Infravermelha (TI) é um método barato, indolor, sem emissão de radiação e de fácil manejo que pode determinar a temperatura da pele do membro a ser amputado, o que auxiliaria, assim, o cirurgião na escolha do melhor nível de amputação.

**Objetivo:**

Avaliar o uso da TI para escolha do nível da amputação em pacientes portadores de DAOP e IC dos MMII. Método: Estudo de Coorte Prospectivo, realizado no período de abril de 2023 a novembro de 2023, na Unidade de Cirurgia Vascular do Hospital de Base do Distrito Federal (Brasília-DF). O estudo incluiu pacientes com DAOP e IC dos MMII candidatos à amputação dos MMII. Todos foram submetidos à TI dos MMII utilizando protocolos padronizados com medidas térmicas em quatro áreas do membro inferior.

**Resultados:**

Os resultados evidenciaram um amplo gradiente térmico longitudinal em pacientes com DAOP e tabagistas (T) em comparação aos não tabagistas (NT). Os pacientes T com gradientes térmicos mais amplos apresentaram maior probabilidade de serem submetidos à uma amputação transfemoral.

**Conclusão:**

A termografia infravermelha é útil tanto em detectar diferenças térmicas em pacientes com DAOP e IC dos MMII, quanto na avaliação não invasiva pré-operatória destes doentes. Pacientes tabagistas com maiores gradientes térmicos longitudinais apresentam maior probabilidade de serem submetidos a amputações transfemorais.

## INTRODUCTION

Peripheral arterial obstructive disease (PAOD) is highly prevalent. Critical ischemia (CI) of the lower limbs (LL) is its final stage and requires surgical treatment that can range from open or endovascular revascularization or partial or total amputation^1^.

Amputation of lower limbs is one of the most devastating consequences for patients with PAOD, and this outcome is associated with some risk factors, such as diabetes mellitus (DM), systemic arterial hypertension (SAH), and smoking. The choice of the best level of amputation in these patients is based on several criteria, the main one still being the surgeon’s clinical judgment. The correct choice implies lower financial costs and lower mortality, since it avoids subsequent surgeries and reduces the length of hospital stay^1^ ^-^ ^3^.

Infrared thermography (IT) is a non-invasive, painless, and low-cost method that captures body temperature and has its use established in several areas of medical sciences. This complementary diagnostic method has been recognized by the American Academy of Medical Infrared Imaging since 1887, and its fundamental principle is the temperature difference between two points, where asymmetries above 0.7º C are considered abnormal and may indicate relevant clinical change^4^ ^-^ ^6^.

In this context, IT has potential utility in detecting thermal reduction in patients with PAOD, especially in those with lower limbs CI. Although some studies have shown its usefulness in the evaluation of patients with obstructive atherosclerosis of the lower limbs, its use as a preoperative instrument to assist in defining the level of amputation has not yet been described^7^ ^-^ ^10^. 

From this perspective, the present study aimed to investigate the usefulness of IT for choosing the level of amputation in patients with PAOD and lower limbs CI.

## METHODS

This was a prospective cohort study conducted from April 2023 to November 2023, at the Vascular Surgery Unit of the Hospital de Base do Distrito Federal, in Brasília, Brazil’s Federal District. We included patients with PAOD and lower limbs CI who were candidates for primary amputation of the lower limbs, admitted to the institution’s emergency room and Vascular Surgery Service ward.

The study was approved by the institution’s Ethics Committee, under CAAE protocol number 64758822.1.0000.5208. We excluded patients with bone tumors, severe infection in the lower limbs, and those undergoing lower limbs open or endovascular revascularization. We also excluded patients who refused to sign the informed consent form (ICF).

For the collection of thermal measurements, we adopted the protocol based on the International Consensus and Guideline for Medical Thermography^11^ ^-^ ^13^. An acclimatization period of 15 minutes was respected, without manipulation of the examined area, and the temperature of the environment was controlled with air conditioning (24ºC ± 2ºC). The collection of thermographic data happened as follows:

1) All infrared thermal images were acquired using a Flir Cx-Series Thermo cam handheld digital camera (FLIR Systems AB, Sweden), with a weight of 130g, a 240x180 infrared sensor (43,200 measurement pixels) and thermal sensitivity > 0.05º C. Thermograms were analyzed using the FLIR ResearchIR^®^ program (FLIR Systems Inc). We considered an altered thermogram one showing a temperature difference greater than 2° C between two points of any lower limb, suggesting a possible level of amputation.
2) Temperature recording (ºC) at four sites of the lower limb to be amputated: forefoot and toes, ankle at the level of the malleolus, 4 cm below the tibial tuberosity, and 4 cm above the patellar tendon. This was considered the study limb.
3) Temperature recording (ºC) at four sites of the lower limb contralateral to the amputation: forefoot and toes, ankle at the level of the malleolus, 4 cm below the tibial tuberosity, and 4 cm above the patellar tendon. This was considered the control limb.
4) Recording of minimum, maximum, and average temperatures (ºC) of both lower limbs.
5) Longitudinal thermal gradient recording: Difference between the minimum and maximum temperature in the limb to be amputated. After the temperature recordings, an amputation level immediately above the necrotic tissue was suggested, with a difference of at least 2º C.

Temperatures and their respective gradients were tabulated, and the level of amputation performed by the vascular surgeon according to the clinical evaluation was checked using the surgical description sheet. All temperature evaluations with IT were performed by a single evaluator, but the surgeries were performed by different physicians who used their own criteria to define the level of amputation and did not have access to the thermographic results.

The sample size was calculated by comparing means between groups of smokers (S) and nonsmokers (NS), with a statistical power of 90%, two-tailed hypothesis test, a minimum expected difference of 2.0º C, and a significance level of 5%. The estimated required sample was 20 patients per group. For the analyses, we stratified patients as S or NS. Continuous variables with a distribution close to normal were expressed as means ± Standard Deviation.

The normality of the data was analyzed visually and using the Shapiro-Wilk test. We used Analysis of covariance (ANCOVA) to evaluate the thermal differences between S and NS patients, with adjustment for the variables age and diabetes mellitus. To compare the levels of the variable ankle brachial index (ABI), the ANCOVA was adjusted for age, diabetes mellitus, and smoking. To assess the degree of agreement between evaluators, we used the Cohen’s Kappa test. Categorical variables were expressed as percentages. A p-value < 0.05 in the two-tailed hypothesis test was defined as statistically significant. Data analysis was performed using the SPSS statistical package, version 26.

## RESULTS

Among the 46 selected participants, 40 met the eligibility criteria and agreed to participate in the study. Among the 40 patients evaluated, 24 (60%) were male. Regarding the risk factors for PAOD, approximately 70% of the patients had SAH, 60% DM, and 50% were smokers (Table 1). 

**Table 1:** Characterization of the sample of patients with critical ischemia, smokers and nonsmokers, submitted to infrared thermography to evaluate the level of amputation.

	Smokers (n=20)	Non-Smokers (n=20)
Age	68,4 (15,2)	74,1 (12,8)
Weight (kg)	66,3 (12,1)	69,9 (9,7)
Height (cm)	167,0 (10,6)	167,3 (9,7)
Sex (male), %	70	50
BMI, %		
<18,5	10	5
18,6 - 24,9	15	35
25,0 - 29,9	75	50
30,0 - 34,9	-	5
35,0 - 39,9	-	5
Race, %		
White	45	55
Black	15	15
Brown	40	25
Yellow	-	5
Marital status, %		
Married	45	40
Single	10	10
Divorced	15	5
Widower	30	40
Civil union	-	5
	Smokers (n=20)	Non-Smokers (n=20)
CRF, %		
No	85	65
DM		
Yes	40	80
ABI, %		
<0,39	30	15
0,4 - 0,59	45	50
0,6 - 0,79	25	25
0,8 - 0,9	-	10
SAH, %		
YES	70	80
WIFI, %		
Low risk	25	30
Moderate risk	30	40
High risk	30	25
Irrecoverable	15	5

**Figure 1:**
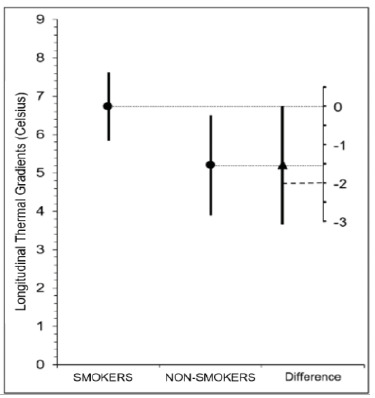
Confidence interval representing the difference for the longitudinal thermal gradients (ºC) of the Experimental limb (amputee) between smokers and nonsmokers (95%CI 0.01-3.06). Thermal variations of the skin greater than 2ºC are pointed out as of greater pathological importance.

Table 2 and Figure 1 show the differences between the thermal means for S and NS patients. The means for the minimum experimental temperature (amputated limb) did not show statistically significant differences between the S and NS groups (p=0.321). 

**Table 2:** Thermal means (SD) and thermal differences (95% CI) between groups of smokers and nonsmokers.

	Smokers	Non-smokers	Differences between means (95% CI)	p-value
Control mean, ºC	34,20 (1,6)	34,32 (1,5)	- 0,12 (-1,11 a 0,87)	0,506
Control min., ºC	32,81 (2,4)	32,55 (2,5)	0,26 (-1,31 a 1,83)	0,748
Control max., ºC	35,50 (1,2)	35,60 (1,1)	- 0,10 (- 0,84 a 0,64)	0,624
Experimental mean, ºC	31,97 (1,7)	33,07 (2,0)	- 1,10 (- 2,29 a 0,09)	0,038
Experimental min., ºC	28,30 (2,0)	30,14 (3,4)	- 1,84 (- 3,63 a 0,05)	0,321
Experimental max., °C	35,11 (1,5)	35,43 (1,2)	- 0,32 (- 1,19 a 0,55)	0,601
∆Mean (E - C), ºC	2,22 (1,1)	1,23 (1,2)	1,00 (0,25 a 1,73)	0,017
∆Experimental (min - max), ºC	6,73 (1,9)	5,20 (2,8)	1,53 (0,01 a 3,06)	0,049

∆: gradient; Min: minimum; Max: maximum; E: experimental (amputated limb); C: control (non-amputated limb). ANCOVA adjusted for the variables Age and Diabetes mellitus.

[Fig f2]

**Figure 2 f2:**
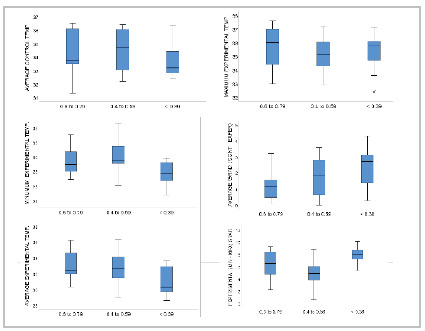
Mean, maximum and minimum (ºC) temperatures measured at different sites in the control (non-amputee) and experimental (amputee) limbs, stratified by the ankle-brachial index. Boxplots display the means (colored rectangles), the medians (horizontal line), interquartile range (size of the rectangles), maximum and minimum values (whiskers), and a moderate outlier (circle). * p = 0.032; ** p = 0.002. ANCOVA adjusted for the variables age, diabetes mellitus, and smoking. Grad: gradient; Min: minimum; Max: maximum.

To evaluate the agreement between the suggested amputation levels and those performed, we applied the Cohen’s Kappa test^14^. In this context, we estimate coefficients of determination (COD) as a percentage of the confidence data. The results suggest moderate agreement for transfemoral (Kappa=0.609; p<0.01; COD=37%) and toes (Kappa=0.684; p<0.01; COD=47%) levels; weak agreement for the Lisfranc level (Kappa=0.474; p=0.003; COD=22%); minimum agreement for the transtibial level (Kappa=0.220; p=0.157; COD=4.8%); and no agreement for the Choppart level (Kappa=0.077; p=0.583; COD=0.6%). 

The percentage agreement between the level of amputation suggested by the vascular surgeon and the level performed varied substantially. For transfemoral amputations, the agreement was 40.0%, toes 10.0%, Lisfranc 7.5%, Transtibial 5.0%, and no agreement for Chopart amputations (0%). The mean lower limb temperatures in S patients were 31.7ºC, while in NS patients it was 33.07 (p=0.038). Table 3 shows the relationship between the frequencies of amputations suggested by IT and those performed at each level.

**Table 3:** Columns representing the percentage of agreement/disagreement between evaluators for different amputated limbs.

Amputation Sugested	Amputation Artelhos	Amputation Lisfranc	Amputation Chopart	Amputation Transtibial	Amputation Transfemoral	Total
Amputation Artelhos	4	2	0	0	0	6
Total %	10,0%	5,0%	0,0%	0,0%	0,0%	15,0%
Amputation Lisfranc	0	3	2	0	0	5
Total %	0,0%	7,5%	5,0%	0,0%	0,0%	12,5%
Amputation Chopart	0	0	0	5	2	7
Total %	0,0%	0,0%	0,0%	5,0%	7,5%	12,5%
Amputation Transtibial	0	0	0	1	6	17
Total %	0,0%	0,0%	0,0%	5,0%	40,0%	42,5%
Total	4	6	2	5	16	33
Total %	10,0%	15,0%	5,0%	12,5%	57,5%	100%

## DISCUSSION

In this study, the risk factors found for PAOD were similar to those already described in the literature, as reported by Czerniecki et. al. in 2022, when describing the main conditions related to the increased chance of contralateral lower limb amputation in patients who had already been amputees^15^.

Our findings suggest that smokers (S) have higher thermal amplitudes as measured by infrared thermography (IT) compared with nonsmokers (NS), especially when estimated by longitudinal thermal gradients, reaching values above 6° C. This result is clinically relevant, since we observed a higher frequency of transfemoral amputations in patients with high longitudinal thermal gradients. Therefore, IT may assist in the identification of more severe scenarios in this specific population. 

It is important to highlight that the thermal differences observed between groups S and NS may have occurred due to patients in group S more frequently developing severe forms of PAOD. These forms are characterized by the simultaneous involvement in multiple arterial sites, including the femoral, leg, and foot arteries.

Ilo A et al. (2020) carried out multiple IT thermal comparisons between feet of diabetic patients and showed a mean difference of about 1.46 ± 1.4º C in the feet with the highest risk of amputation. From this perspective, it is likely that even thermal gradients below the 2º C threshold, as observed in the present study, are also of clinical importance for the screening of patients at higher risk of amputation, particularly when there are other associated prognostic factors, such as smoking^16^. 

As for the agreement between the amputation levels suggested by IT and those performed by the vascular surgeons, we observed moderate to no agreement. Although some Kappa test results have indicated statistically significant differences, the strength of the evidence is still insufficient to recommend amputations based solely on IT. This is one of the reasons why many experts recommend a minimum acceptable agreement of at least 80%^12^, especially when the intervention involves important changes in medical conduct. This may have occurred because the levels suggested by IT consider only the thermal gradient between the different levels of amputation (Transfemoral, Transtibial, Chopart, Lisfranc, and toe), and no other factors. In this case, we used a minimum longitudinal thermal gradient of 2º C to suggest the level of amputation. 

The results presented suggest a tendency on the part of vascular surgeons to perform transfemoral amputations, even when the thermal gradient advise that amputations at other levels could be more appropriate. However, the reasons that lead these professionals to opt for this type of amputation are the most varied, especially the clinical judgment based on the doctor’s previous experience (subjective probability). In addition, other factors, such as chronic and irreversible immobility, the presence of ankylosis of the knee joint, high cardiovascular risk, and the low probability of using orthopedic prostheses, may also influence the decision for the level of amputation.

It is worth noting that vascular Doppler ultrasound is an auxiliary method in determining the level of amputation. Nonetheless, this imaging method is observer-dependent and is subject to the typical biases related to the examiner’s experience. This can render the interpretation challenging, especially in the evaluation of sequential and high-grade strictures^16^ ^-^ ^19^. 

The ideal test for determining the level of amputation should be practical, low-cost, and with good sensitivity and specificity. The literature is still lacking in controlled clinical studies that can reliably predict the level of amputation. More accurate tests require higher costs, which is not always reproducible in clinical practice. The comparison with other more accurate exams, such as arteriography of the lower limbs, could provide more comparative information related to the best level of amputation. However, the patients who underwent primary amputation were not submitted to more invasive exams or the use of iodinated contrast agent, since they had a low probability of supporting a satisfactory arterial revascularization. The non-comparison of the results between IT and other vascular diagnostic methods was a limitation of the present study. Future studies may bring encouraging results by comparing IT with the gold standard method in the diagnosis of PAOD.

From this perspective, one option would be to use a test that, although not as accurate, would be cheaper and display better clinical application. Thus, IT, performed at the bedside, is a practical, painless, low-cost method that can provide important information for the surgeon in determining the best level of amputation. We carried out no comparison of the financial cost of IT with another complementary method, so the considerations made on this aspect are based on data from the literature.

The use of IT is not new and despite its promising results, further studies are needed for its validation as a useful tool in the evaluation of patients with PAOD, as well as a larger number of patients, including stratifying into other risk groups such as diabetes mellitus and hypertension. Despite the limitations, it is possible to speculate that the use of IT could aid surgeons’ decision-making when defining the level of amputation in advanced stages of PAOD.

One limitation of the present research was that it did not use dynamic IT. This is a promising method for analyzing the blood flow of the extremity of the affected limb and may be an interesting tool in the diagnosis and treatment of patients with PAOD. We also did not compare IT with Doppler ultrasound, since the latter was not always available to be performed by the vascular surgeon who carried out the amputation. Another limitation of the research was the comparison with late postoperative results, especially re-amputation rates. Measurements of residual stump temperatures may be clinically important for these patients, especially candidates for prosthetic rehabilitation.

## CONCLUSION

Patients with peripheral arterial occlusive disease and smokers had greater longitudinal thermal gradients when compared with nonsmokers. These results are of clinical importance, since these patients underwent transfemoral amputations more frequently. Although the means for the minimum experimental temperature (amputated limb) did not show statistically significant differences between the groups of smokers and nonsmokers, the results of the 95% confidence interval suggest that these differences may be greater than 3º C.

There was a greater agreement between the level of amputation suggested by thermography and that indicated by the surgeon for transfemoral (40%), toe (10%) and Lisfranc (7.5%) amputations. IT can be a complementary method in the evaluation of the level of amputation in patients with critical ischemia of the lower limbs.
